# Synergistic Antifungal Activity of Chito-Oligosaccharides and Commercial Antifungals on Biofilms of Clinical *Candida* Isolates

**DOI:** 10.3390/jof7090718

**Published:** 2021-09-01

**Authors:** Monica Ganan, Silje B. Lorentzen, Peter Gaustad, Morten Sørlie

**Affiliations:** 1 Department of Chemistry, Biotechnology, and Food Science, Norwegian University of Life Sciences (NMBU), 1432 Aas, Norway; monicaganan@gmail.com (M.G.); Silje.lorentzen@nmbu.no (S.B.L.); 2 Department of Microbiology, Institute of Clinical Medicine, University of Oslo and Fürst Medical Laboratory, 0154 Oslo, Norway; peter.gaustad@medisin.uio.no

**Keywords:** chito-oligosaccharides, chitosan, antifungal, yeast, *Candida*, biofilm

## Abstract

The development of yeast biofilms is a major problem due to their increased antifungal resistance, which leads to persistent infections with severe clinical implications. The high antifungal activity of well-characterized chitosan polymers makes them potential alternatives for treating yeast biofilms. The activity of a chito-oligosaccharide with a depolymerization degree (DP_n_) of 32 (C32) and a fraction of acetylation (F_A_) of 0.15 on *Candida* sp. biofilms was studied. The results showed a concentration-dependent reduction in the number of viable cells present in *C. albicans, C. glabrata*, and *C. guillermondii* preformed biofilms in the presence of C32, especially on intermediate and mature biofilms. A significant decrease in the metabolic activity of yeast biofilms treated with C32 was also observed. The antifungals fluconazole (Flu) and miconazole (Mcz) decreased the number of viable cells in preformed early biofilms, but not in the intermediate or mature biofilms. Contrary to Flu or Mcz, C32 also reduced the formation of new biofilms. Interestingly, a synergistic effect on yeast biofilm was observed when C32 and Flu/Mcz were used in combination. C32 has the potential to become an alternative therapeutic agent against Candida biofilms alone or in combination with antifungal drugs and this will reduce the use of antifungals and decrease antifungal resistance.

## 1. Introduction

Modern medicine is challenged by an increasing resistance in *Candida* spp to antifungals [1]. There is a need for new treatment options to overcome the problem and the development of new antifungals as well as the use of combination therapy. Chitosan as well as oligomers of chitosan known as chito-oligosaccharides (CHOS) have an antifungal effect in in vitro experiments [2].

In vitro studies have also shown that there is a synergistic, antifungal effect of the combination of commercial antifungals (CA) and chitosan as well of the oligomers CHOS [3,4]. The use of CHOS and in synergistic combination with CA will, if used for treatments of fungal infections in the future, reduce the number of antifungals needed for treatment, decrease the development of antifungal resistance, and improve the treatment of resistant fungi.

A biofilm is a microbial community and a protected niche for microorganisms. Many fungi are capable of biofilm growth. The significance of this growth is that biofilm formation can occur during many kinds of infections, and biofilm growth is a major cause of recurrent infections. Biofilms have limited drug susceptibility, making infections difficult to treat. Biofilm formation proceeds through three distinct developmental phases: early, intermediate, and mature. During these phases, yeasts progress from adherent blastospores to well-defined cellular communities encased in a polysaccharide matrix [5].

The development of pathogenic biofilms remains a major problem with severe clinical implications. *Candida* spp. are pathogenic and can cause both superficial and serious systemic disease. Many *Candida* systemic infections involve the formation of biofilms on implanted devices such as indwelling catheters, joint replacements, or prosthetic heart valves [6,7]. Additionally, *Candida* biofilms have also been related to topical infections such as vaginal candidiasis [8,9] and oral candidiasis [10]. Candida biofilms are resistant to a range of antifungal agents currently in clinical use including amphotericin B, nystatin, and azoles including the newer triazoles, and have reduced antifungal activity of lipid formulations of amphotericin B and echinocandins. Multiple resistance mechanisms are involved [11].

Chitosan is a cationic linear copolymer composed of a variable number of β-(1–4) linked units of 2-acetamide-2-deoxy-β-d-glucopyranose (GlcNAc) and 2-amino-2-deoxy-β-d-glycopyranose (GlcN) [12], obtained by the alkaline deacetylation of chitin. Properties such as the fraction of acetylation (F_A_), and the degree of polymerization (DP) are fundamental for the physical-chemical properties of the chitosan polymer including solubility and conformation [13,14,15]. The use of oligomers of chitosan, also known as chito-oligosaccharides (CHOS), allows for conquering the reduced solubility of chitosan in water at pH 7.

The singular properties of chitosan and its polymers make them well-suited for a wide range of biomedical applications such as drug delivery [16,17,18], immunoprophylaxis, wound healing, neural stem cell growth, tissue engineering, and gene therapy [19]. These properties include biodegradability in the human body and low cytotoxicity, which confer chitosan good biocompatibility characteristics [20].

However, possibly one of the most interesting features of the chitosan polymer CHOS is its antimicrobial and antifungal activity against a wide range of bacteria, yeasts, and molds [2,21]. Antimicrobial activity is also known from a variety of peptides and their synergy with antimicrobial agents have been studied [22,23]. Nevertheless, the effects of CHOS and antimicrobial peptides on biofilms remain understudied and the few studies published are not quantitative. The aim of this preliminary work was to quantitatively study the effect of a well-characterized CHOS alone and in combination with commercial antifungals on *Candida* biofilms of clinical isolates to explore its potential to be used in antifungal therapy and for the prevention and treatment of fungal biofilms on indwelling medical devices and superficial infections.

## 2. Materials and Methods

### 2.1. Enzymatic Production of Chito-Oligosaccharides

CHOS with DP_n_ of 32 and *F*_A_ of 0.15 as determined by ^1^H-NMR (abbreviated C32) was prepared as described previously [4,24]. Briefly, chitosan (KitoNor, Norwegian *Chitosan*, Gardermoen, Norway, FA 0.15, DPn 206) was obtained from Norwegian Chitosan, Gardermoen, Norway. Chitosan (10 mg mL^−1^) in 0.04 M NaAc, 0.1 M NaCl, 1% HCl was incubated at 37 °C and 225 rpm until the chitosan was dissolved (approximately 15 min). The pH was then adjusted to 5.5 with 0.5 M NaOH. Chitosanase *Sc*Csn46A (0.5 μg per mg chitosan) from *Streptomyces coelicolor* (UniProt accession code q9rj88) was added to the solution and the mixture was incubated for 20 min at 37 °C and 225 rpm. The enzymatic reaction was stopped by decreasing the pH to 2.5 with HCl (0.5 M), followed by immersing the tube in boiling water for at least 10 min to inactivate the enzymes permanently. The resulting CHOS sample was dialyzed against dH_2_O for 48 h (water was changed every 12 h) using a cellulose membrane (Float-A-Lyzer MWCO 500 Da, from Spectrum Labs, Arlington, TX, USA) to remove buffer salts from the sample. Dialyzed samples were sterile filtrated through Filtropur S 0.2 μm sterile filters (Sarstedt, Nümbrecht, Germany), lyophilized, and stored at 4 °C.

### 2.2. Commercial Antifungals (CA)

The triazole fluconazole (Flu) and azole miconazole (Mcz) were purchased from Sigma (St. Louis, MO, USA). Mcz is mainly used in the local treatment of superficial fungal infections while Flu can be used for superficial and deep infections.

### 2.3. Candida Strains

Clinical isolates from patients with infections of *Candida albicans* (1581), *Candida parapsilosis* (27,470), *Candida glabrata* (3808), and *Candida guillermondii* (12,146) from the Oslo University Hospital strain collection were used for the experiments. *C. albicans* and *C. glabrata* strains were included in all the experiments, while *C. parapsilosis* was used in biofilm formation experiments and *C. guillermondii* was also included in the study on the preformed biofilms. The two strains *C. albicans* and *C. glabrata* were chosen because they represent susceptibility and resistance to Flu and Mcz.

### 2.4. Candida Inocula

*Candida* strains were grown on Sabouraud agar at 37 °C for 48 h. Yeast suspensions were prepared in sterile water by touching ten colonies from a culture plate and standardized to 1 × 10^7^ CFU mL^−1^) using spectrophotometric methods. One milliliter of the fungal suspension was added to 9 mL of Roswell Park Memorial Institute Medium (RPMI, Merck KGaA, Darmstadt, Germany).

### 2.5. Biofilm Formation

Biofilm production was based on the method standardized by Chandra et al. (2001) with some modifications [5]. Briefly, yeast inocula were seeded into the wells (1 mL well^−1^) of pre-sterilized, polystyrene, flat-bottom, tissue culture-treated, 24-well microtiter plates with a 1.9 cm^2^ well surface (Corning Incorporated, Corning, NY, USA). Non-adherent cells were removed by washing the wells three times in sterile PBS, and RPMI medium was added (1 mL well^−1^). Microtiter plates were then incubated for 3, 24, and 48 h at 37 °C.

### 2.6. Candida Cell Enumeration in Biofilms

Biofilm disruption was made by trypsinization as described next. Biofilms were incubated at 37 °C with 200 μL well^−1^ of Trypsin-EDTA solution (0.05% trypsin, 0.02% EDTA) for 3 min. Incubation of yeasts with the Trypsin-EDTA solution did not affect the viability of the yeasts. After incubation, 800 μL well^−1^ of RPMI was added and biofilms were disrupted by scraping. The content of each well was then transferred to an Eppendorf tube and vortexed vigorously for 30 s. Tubes were centrifuged at 5000 rpm for 10 min and washed three times in PBS. Yeasts were enumerated by serial dilution and seeding in Sabouraud agar.

### 2.7. Metabolic Activity Determination

After biofilm formation, the medium was aspirated, and non-adherent cells were removed by washing the wells three times in sterile PBS. Afterward, 1 mL of PBS, 10 μL menadione solution (1 mM in DMSO), and 12.5 μL 2,3-bis(2-methoxy-4-nitro-5-sulfophenyl)-5-[(phenyl amino) carbonyl]-2H-tetrazolium hydroxide (XTT) were added to each well [25]. Plates were then incubated at 37 °C for 5 h. After incubation, the content of the wells was transferred into tubes and centrifuged (5 min, 5000 rpm). XTT formazan in the supernatant was determined spectrophotometrically (492 nm).

### 2.8. Effect of C32 and Flu on Preformed Biofilms

Working concentrations of C32 and Flu were prepared in RPMI using a 20 or 12.8 mg mL^−1^ stock solution, respectively. *Candida* biofilms, obtained as described above, were washed three times with sterile PBS to remove non-adherent cells. C32 or Flu in RPMI, at different concentrations, were added to the wells (1 mL well^−1^). The plates were then incubated for 24 h at 37 °C. The effect of C32 and Flu on preformed biofilms was estimated by plate counting, as previously described.

### 2.9. Effect of C32 and CA on Biofilm Formation

Yeast inocula were prepared in sterile water as previously described. One milliliter of the suspension was diluted into 9 mL of C32 or commercial antifungals (CA) in RPMI at the appropriate concentration and then added to the microtiter wells (1 mL well^−1^). Microtiter plates were incubated for 48 h at 37 °C. *Candida* biofilm enumeration was performed as described above after 3, 24, and 48 h of incubation. Concentrations were selected based on previous studies performed by our group on planktonic *C. albicans* and *C. glabrata* cells. The sub-inhibitory concentrations used in the *C. glabrata* experiments were 5000, 32, and 6.25 μg mL^−1^ for C32, Flu, and Mcz, respectively, and in the *C. albicans* experiments 5000, 0.12, and 16 μg mL^−1^, respectively.

### 2.10. Synergy

Ranges of minimal detaching concentrations (MDC) for each antifungal were assessed. MDC was the lowest drug concentration that reduced ≥ 1 log the biofilm counts after 24 h of incubation at 37 °C. Synergy tests were performed in duplicate using combinations of C32 and Flu or Mcz. Positive growth controls were performed in wells not containing antifungals. Minimal detaching concentration in combination (MDCC) was the lowest concentration of the antifungal when used in combination, which reduced ≥ 1 log yeast attachment after 24 h of incubation at 37 °C. Fractional detaching concentration (FDC) index was the MDCC of each drug divided by the MDC. FDC summation (∑FDC) of each antifungal combination was calculated for every strain tested. Summation values were interpreted as follows: ≤ 0.5, synergy (S), > 0.5 and ≤ 4: indifference (I), > 4: antagonism (A).

### 2.11. Statistical Analysis of Data

Experiments were done at least in duplicate. Experimental data were analyzed using Minitab version 14.1 (Minitab 14, State College, PA, USA). Student’s *t-*tests were performed to identify differences between samples. Differences were significant when *p* ≤ 0.05. Tukey’s range test was used to assess differences between pairs of means.

## 3. Results

### 3.1. Candida Biofilm Formation

**Figure 1:**
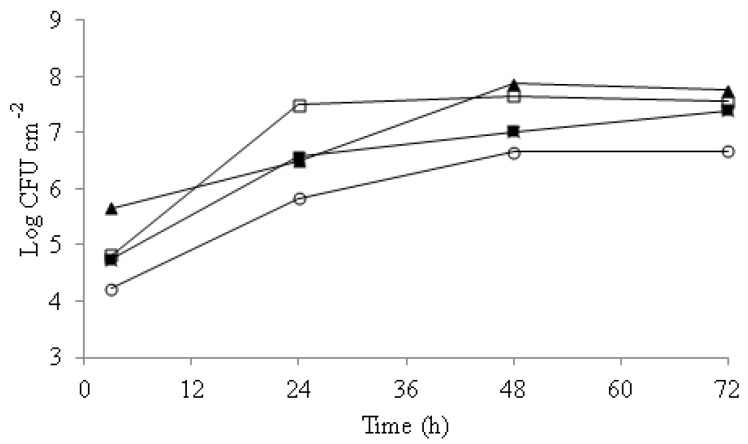
Biofilm counts along 72 h incubation time. *C. albicans* (□), *C. guillermondii* (▲), *C. glabrata* (■), *C. parasilopsis* (○). Experiments were performed in duplicate and the displayed results are from a representative experiment.

Biofilm formation ability was studied for 72 h on *C. albicans, C. glabrata*, *C. parasilopsis,* and *C. guillermondii* (Figure 1).

Differences in attachment were observed among strains along the incubation time. *C. guillermondii* was the strain with the highest initial attachment to the wells, with 5.7 Log CFU cm^−2^, followed by *C. albicans* and *C. glabrata*, both with 4.8 Log CFU cm^−2^, and 4.1 Log CFU cm^−2^ in the case of *C. parasilopsis*. After 24 h of incubation, significant increases in the *Candida* spp counts were recorded with populations of 7.6 Log CFU cm^−2^ for *C. albicans*, 6.5 Log CFU cm^−2^ for *C. guillermondii*, and 5.8 Log CFU cm^−2^ for *C. parasilopsis.* Between 48 and 72 h of incubation, the biofilm yeast counts did not change significantly (*p >* 0.05).

### 3.2. Effect of C32 and CA on Preformed Candida Biofilms

**Figure 2:**
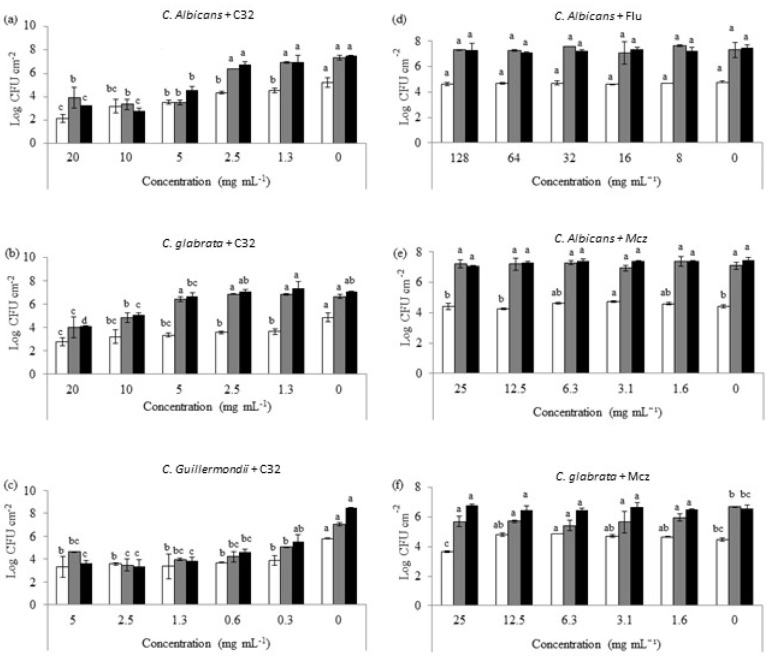
*Candida* cell counts of early (3 h, white), intermediate (24 h, grey), and mature (48 h, black) biofilms exposed to different concentrations of C32 (*C. albicans* (**a**), *C. glabrata* (**b**), *C. guillermondii* (**c**)) and antifungals (*C.* albicans and Flu 0.12 μg mL^−1^ (**d**), C. albicans and Mcz 16 μg mL^−1^ (**e**), C. glabrata and 6.25 μg mL^−1^ Mcz (**f**)). Bars with the same color and different letters were statistically different (*p* ≤ 0.05). Experiments were performed in triplicate and the results are displayed as the mean of each individual experiment with the calculated standard deviation shown as error bars.

The potential of C32 to be used for detached preformed yeast biofilms was studied on *C. albicans, C. glabrata,* and *C. guillermondii* (Figure 2) on three different biofilm developmental phases: early (3 h), intermediate (24 h), and mature (48 h).

A concentration-dependent effect on the ability of C32 to reduce biofilm yeast counts was observed. In the case of *C. albicans*, a decrease (*p >* 0.05) in the counts on early biofilms (4.55 Log CFU cm^−2^) when in the presence of 1.3 mg mL^−1^ of C32 compared to the control (5.20 log CFU cm^−2^) was observed. In addition, there was a significant reduction (*p >* 0.05) in the biofilm-forming yeast counts after 24 and 48 h of incubation with C32 (3.49 and 4.58 log CFU cm^−2^, respectively) at 5 mg mL^−1^ when compared to the control (7.34 and 7.48 log CFU cm^−2^, respectively). Similarly, a significant reduction (*p >* 0.05) in the counts (3.64 log CFU cm^−2^) was obtained for *C. glabrata* after 3 h of incubation with 1.3 mg mL^−1^ C32 compared to the control (4.88 Log CFU cm^−2^). After 24 h of incubation, significant reductions (*p >* 0.05) were obtained for concentrations of 10 and 20 mg mL^−1^ C32 (4.85 and 4.02 Log CFU cm^−2^, respectively) compared to the control (6.65 Log CFU cm^−2^). Likewise, after 48 h of incubation, reductions (*p >* 0.05) were detected for concentrations of 5 mg mL^−1^ or higher when compared to the control (6.69 and 7.07 Log CFU cm^−2^, respectively). In the case of *C. guillermondii*, biofilms exposed to 0.3 mg mL^−1^ of C32 showed a significant reduction (*p >* 0.05) compared to the control after 3 h (3.89 and 5.81Log CFU cm^−2^) and 24 h of incubation (5.06 and 7.04 Log CFU cm^−2^). After 48 h of incubation, concentrations of 0.6 mg mL^−1^ significantly (*p >* 0.05) decreased the counts from 8.48 to 4.60 log CFU cm^−2^.

Additionally, the effect of Flu and Mcz on preformed *Candida* biofilms was studied (Figure 2). Flu at the concentrations tested did not show any significant (*p* ≤ 0.05) effect on *C. albicans* biofilms. Flu at concentrations ≥ 32 μg mL^−^^1^ significantly (*p* ≤ 0.05) reduced *C. albicans* counts on early biofilms. However, it did not affect the intermediate or mature biofilms. The highest concentration of Mcz tested (25 μg mL^−1^) significantly (*p* ≤ 0.05) reduced 3 h of old *C. glabrata* biofilms. However, it did not show any effect on 24 or 48 h old biofilms.

### 3.3. Effect of C32 on the Metabolic Activity of Candida Biofilms

**Figure 3:**
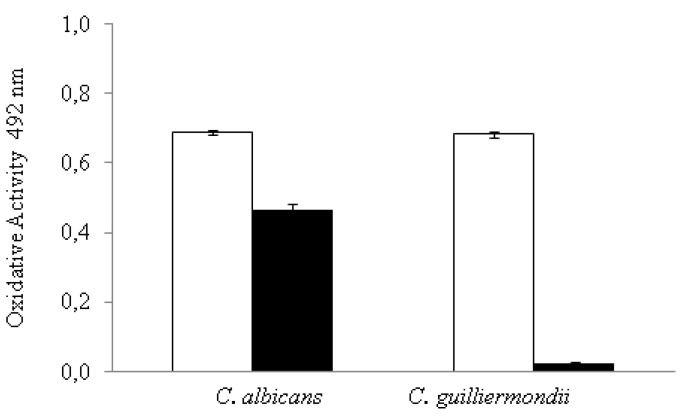
Effect of C32 (black bars) on the metabolic activity of *C. albicans* and *C. guillermondii* (white bars) intermediate biofilms as measured by the absorbance of formed formazan as detailed by Hawser et al. [25], the figure is created in this work. Observed effects were statistically different from the control (*p* ≤ 0.05). Experiments were performed in triplicate and the results are displayed as the mean of each individual experiment with the calculated standard deviation shown as error bars.

A XTT assay was performed, and the absorbance of formed formazan product was determined to measure the metabolic activity of *C. albicans* and *C. guillermondii* on 24 h-old biofilms in the presence and absence of C32 (Figure 3).

Both strains showed a significant decrease in their metabolic activity when treated with C32 compared to the control. However, the decrease was significantly (*p* ≤ 0.05) higher in *C. guillermondii* (0.68 to 0.02 oxidative activity) than in *C. albicans* biofilms (0.69 to 0.46 oxidative activity).

### 3.4. Effect of C32 on the Formation of Candida Biofilms

**Figure 4:**
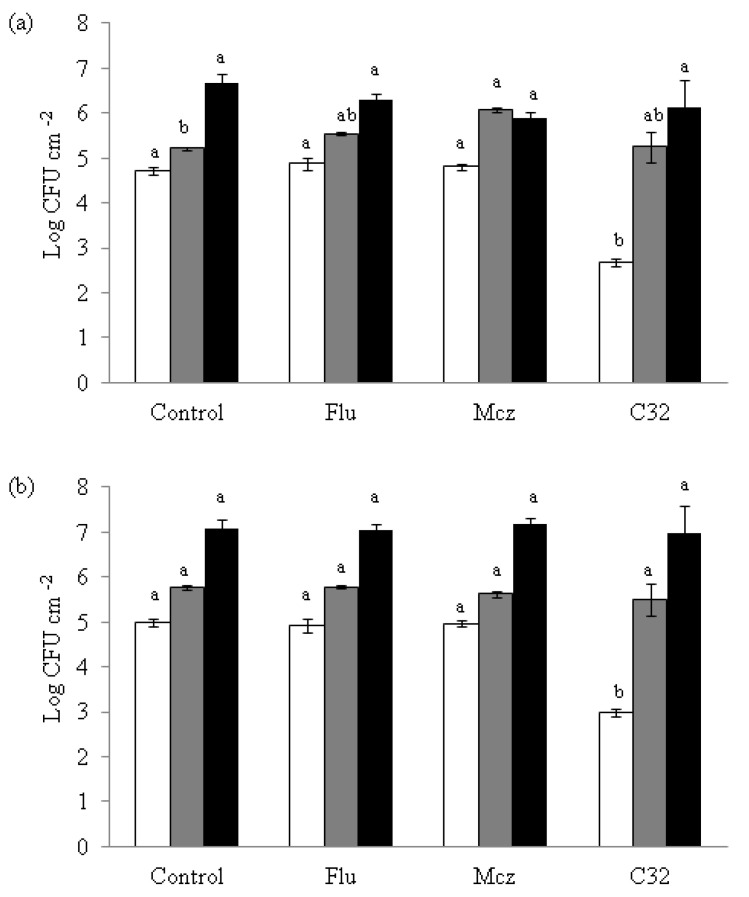
Effect of C32, Flu and Mcz on the formation of *C. albicans* (**a**) and *C. glabrata* (**b**) biofilms after 3 h in accordance with the protocol described by White et al. [26]. The figure is created in this work, 24 h (grey), and 48 h (black) of incubation. Bars with same color and different letters are statistically different (*p* ≤ 0.05). The sub-inhibitory concentrations used in the *C. glabrata* experiments were 5000, 32, and 6.25 μg mL^−1^ for C32, Flu, and Mcz, respectively, and in the *C. albicans* experiments 5000, 0.12, and 16 μg mL^−1^, respectively. Experiments were performed in triplicate and the results are displayed as the mean of each individual experiment with the calculated standard deviation shown as error bars.

The effect of C32 and CA on *C. albicans* and *C. glabrata* biofilm formation was studied (Figure 4).

On both *Candida* strains, samples exposed to C32 showed a significant (*p* ≤ 0.05) lower attachment to the well surfaces (2.67 log CFU cm^−2^ for *C. albicans* and 2.99 log CFU cm^−2^ for *C. glabrata*) at 3 h of incubation than the controls (4.72 log CFU cm^−2^ for *C. albicans* and 4.98 log CFU cm^−2^ for *C. glabrata*). This lower attachment effect was not detected when Flu or Mcz were used not combined with CHOS. At 24 and 48 h, no differences (*p* > 0.05) in yeast attachment were observed compared to the control for any of the compounds tested.

### 3.5. Combined Antifungal Effect of C32 and CA on Preformed Candida Biofilms

The combined effect of C32 and Flu or Mcz was studied on *C. albicans* and *C. glabrata* biofilms to detect possible synergistic effects (Table 1). MDC of C32 and of the antifungals were reduced when used in combination, suggesting a synergistic effect. In the case of *C. albicans*, Flu MDC on early biofilms decreased from 32 to 8 μg mL^−1^ when in the presence of C32, while C32 MDC was reduced from 5000 to 625 μg mL^−1^ when used in combination with Flu.

## 4. Discussion

Pathogenic fungi in the genus *Candida* cause superficial biofilms and biofilms in serious systemic disease. *Candida* spp are involved in the formation of biofilms on implanted devices. The development of pathogenic biofilms remains a major problem with severe clinical implications and therapeutic challenges of antifungal resistance. In this preliminary work, the activity of the well-characterized CHOS C32 on *Candida* biofilms were studied. It was observed that C32 reduced biofilm formation in early developmental stages. Additionally, a reduction in the number of viable cells present in *Candida* preformed biofilms in the presence of C32 was detected, especially in intermediate and mature biofilms. In contrast, it was observed that the azoles Flu and Mcz reduced the number of viable cells in early biofilms, but not in intermediate or mature ones. Concerning these results, in a previous study, Chandra et al. [5] reported that antifungal resistance of biofilm-grown cells increased with the biofilm phase, with mature ones the most resistant to different antifungals including amphotericin B, fluconazole, nystatin, and chlorhexidine.

Previously, chitosan has been tested against biofilms. For instance, using the semi quantitative XXT test, Silva-Dias et al. (2014) [27] found that a low molecular weight chitosan hydrogel impairs biofilm formation and disorganizes preformed ones in vitro and in vivo. Additionally, Martinez et al. [28] showed that chitosan significantly reduced the cell viability and the metabolic activity of *Cryptococcus neoformans* preformed mature biofilms. Using confocal and scanning electron microscopy, the authors also demonstrated that chitosan penetrates biofilms and damages fungal cells. Similarly, Carlson et al. [29] observed reductions in biofilms where viable cell numbers ranged from 95% to 99% on the bacteria *Staphylococcus epidermidis, Staphylococcus aureus, Klebsiella pneumoniae, Pseudomonas aeruginosa*, and on the yeast *Candida albicans* on chitosan-coated surfaces. In the case of *C. albicans*, chitosan-coated surfaces reduced surface-associated growth in more than 3 log CFU cm^−2^ compared to the control surface. Additionally, Tan et al. [30] studied the effect of gentamicin, chitosan, and a chitosan derivative (hydroxypropyltrimethyl ammonium chloride chitosan, HACC) on the formation of *Staphylococcus* spp. biofilms on the surface of bone cements. The group observed that, in contrast to chitosan, HACC prevents bacterial biofilm formation and downregulates virulence-associated gene expression of antibiotic-resistant bacteria. Gentamicin, on the other hand, decreased the number of viable methicillin-resistant Staphylococcus strains, but its ability to inhibit biofilm formation was lower than that of HACC.

Chitosan and its derivatives have, due to their antifungal activity, potential to be used for treating yeast infections in humans [1]. Synergistic drug combinations and combinations of CHOS and antifungals have been shown to be effective against planktonic *Candida* and appear to be potential therapeutic interventions to treat or prevent biofilms [31].

In this study, a reduction in the MDC of both C32 and Flu/Mcz, when used in combination on preformed yeast biofilms, was observed (Figure 2). This finding suggests that the mechanisms of action is a synergistic (Table 1), concentration-dependent reduction in the number of viable cells present in *C. albicans*, *C. glabrata*, and *C. guillermondii* preformed biofilms in the presence of C32, especially on intermediate and mature biofilms. A significant decrease in the metabolic activity of *Candida* biofilms treated with C32 was also observed (Figure 3). The azole antifungals fluconazole (Flu) and miconazole (Mcz) decreased the number of viable cells in preformed early biofilms, but not in intermediate or mature biofilms. Contrary to Flu or Mcz, C32 also reduced the formation of new biofilms. Similar results have been reported by Chauhan et al. [32], who studied the synergistic activity of a chelator (EDTA) and an antibiotic drug. The group showed that a single-dose of EDTA-gentamicin fully eliminated preformed *S. aureus* catheter-associated biofilms in rats. However, EDTA and gentamicin alone were not completely effective against the biofilms. It is known that metal cations are essential for microbial adherence, biofilm formation, and microbial growth. Therefore, metal-binding chelators have the capability of inhibiting biofilm formation by disrupting surface adherence and preventing biofilm production [33]. Similarly to EDTA, chitosan is an important metal-chelator [34], which could explain its detrimental activity on biofilm formation and the synergy observed with antifungal drugs. Using C32 or other non-antifungal drugs in combination with CA will be a way to overcome antifungal resistance in biofilm infections. The mechanism of action studied using confocal imaging showed that C32 adsorbs to the cell surface, with subsequent cell disruption and accumulation of C32 in the cytoplasm [2]. In addition, the use of such combination therapy will reduce the amounts of antifungals used and thus decrease the development of antifungal resistance.

## 5. Conclusions

We conclude that C32 effectively reduced *Candida* biofilms and combined use with the antifungal drugs fluconazole and miconazole had a synergistic effect on *Candida* biofilms. The use of combination therapy with C32 and fluconazole and miconazole will reduce antifungal use and decrease antifungal resistance compared to combination therapy using monotherapy with antifungal drugs. Considering these results, we believe that C32 deserves further exploration for use in clinical practice for the treatment of *Candida* biofilm infections.

## Figures and Tables

**Table 1:** Combined effect of C32 and CA on the growth of Candida biofilms. MDC: minimum detaching concentration (concentrations in μg mL^−1^), CA: commercial antifungals, Flu: fluconazole, Mcz: miconazole, S: synergy, I: indifference.

	CA	Time (h)	CA MDC Alone	CA MDC in Presence of C32	C32 MDC Alone	C32 MDC in Presence of CA	Detection of S or I
*C. albicans*	Flu	3	>32.00	8.00	5000.00	625.00	S
		24	>32.00	8.00	2500.00	625.00	S
		48	>32.00	8.00	2500.00	625.00	S
	Mcz	3	>25.00	6.25	5000.00	625.00	S
		24	>25.00	6.25	2500.00	625.00	S
		48	>25.00	6.25	2500.00	625.00	S
*C. glabrata*	Mcz	3	>25.00	6.25	>10,000.00	625.00	S
		24	>25.00	6.25	10,000.00	625.00	S
		48	>25.00	12.50	10,000.00	1250.00	I
